# Coercivity Mechanism and Magnetization Reversal in Anisotropic Ce-(Y)-Pr-Fe-B Films

**DOI:** 10.3390/ma14164680

**Published:** 2021-08-19

**Authors:** Jun Ma, Xiaotian Zhao, Wei Liu, Yang Li, Long Liu, Yuhang Song, Yuanhua Xie, Xinguo Zhao, Zhidong Zhang

**Affiliations:** 1 Shenyang National Laboratory for Materials Science, Institute of Metal Research, Chinese Academy of Sciences, Shenyang 110016, China; jma17s@imr.ac.cn (J.M.); xtzhao@imr.ac.cn (X.Z.); yli17b@imr.ac.cn (Y.L.); lliu16s@imr.ac.cn (L.L.); yhsong16b@imr.ac.cn (Y.S.); xgzhao@imr.ac.cn (X.Z.); zdzhang@imr.ac.cn (Z.Z.); 2 School of Materials Science and Engineering, University of Science and Technology of China, Shenyang 110016, China; 3 School of Mechanical Engineering and Automation, Northeastern University, Shenyang 110004, China; yhxie@mail.neu.edu.cn

**Keywords:** magnetic domain, magnetization reversal, coercivity mechanism, microstructure, magnetic properties

## Abstract

In this study, the magnetic properties, coercivity mechanism, and magnetization reversal process were investigated for Ce-(Y)-Pr-Fe-B films. After the addition of Y and subsequent heating treatment, the formations of REO (RE ≡ Ce and Pr) and REFe_2_ (RE ≡ rare earths) phases are inhibited, and the microstructure of Ce-Y-Pr-Fe-B film is optimized. Meanwhile, the coercivity and the squareness of the hysteresis loop are significantly improved. The coercivity mechanism of Ce-Y-Pr-Fe-B film is determined to be a mixture of nucleation and pinning mechanisms, but dominated by the nucleation mechanism. The demagnetization results show that the nucleation of reversal magnetic domains leads to irreversible reversal. Our results are helpful to understand the coercivity mechanism and magnetization reversal of permanent magnetic films with multi-main phases.

## 1. Introduction

Rare earth (RE) permanent magnets, especially, Nd-Fe-B based ones [1], have been widely applied in energy and information fields [2] due to their excellent magnetic properties [3]. The intrinsic magnetic properties (including saturation magnetization, anisotropy field at room temperature, and Curie temperature) of Pr_2_Fe_14_B compound are similar to those of Nd_2_Fe_14_B. However, Nd_2_Fe_14_B undergoes a spin reorientation at 135 K [4], and the easy magnetization direction of Pr_2_Fe_14_B is along the c-axis during the whole temperature range. Therefore, Pr_2_Fe_14_B is more suitable for practical applications and studying the coercivity mechanism at a wide range of temperatures. In recent years, with the development of RE, permanent magnetic materials, Pr and Nd resources, have become more and more scarce [5,6], resulting in a massive accumulation of associated high-abundance RE (La/Ce/Y); meanwhile, the use of high-abundance RE could reduce the cost of permanent magnets.

As the most abundant RE element in the Earth’s crust, the structure and magnetic properties of R-Fe-B with Ce have been investigated [7,8,9]. When Pr or Nd is replaced by Ce, the intrinsic magnetic properties of Ce_2_Fe_14_B (μ_0_H_a_ = 2.6 T, J_s_ = 1.17 T) are far inferior to those of Pr_2_Fe_14_B (μ_0_H_a_ = 8.7 T, J_s_ = 1.56 T) and Nd_2_Fe_14_B (μ_0_H_a_ = 6.7 T, J_s_ = 1.60 T), and therefore its coercivity decreases significantly [10]. In order to reduce the magnetic dilution effect, multi-main-phase sintered magnets have been used to prepare magnets with good performance [11]. Moreover, when a large amount of Ce was used, CeFe_2_ phase appeared [12], which required a further heat treatment to adjust the microstructure of the magnet to improve magnetic properties [13]. The Y element is another high-abundance RE that can be used for multi-main-phase magnets. On the one hand, the Curie temperature of Y_2_Fe_14_B phase is slightly higher than that of Ce_2_Fe_14_B phase [4]. The thermal stability of the magnet can be improved by adding Y [14]. On the other hand, due to the negative substitution energy [15], Ce is easier to enter the grain boundary phase than Pr, while Y prefers to exist in the main phase of RE_2_Fe_14_B rather than Pr [16]. The addition of Y affects the distribution of elements [17] and can also reduce the formation of CeFe_2_ phase, therefore, the microstructure of the magnet can be optimized after corresponding heat treatment [14]. 

For Pr-Fe-B magnets, current studies have mainly focused on improving magnetic properties [18]. Depending on preparation methods and components [19,20,21], there are mainly two types of coercivity mechanism of RE-Fe-B magnets, namely pinning and nucleation. For anisotropic Pr-Fe-B films, nucleation is the dominant mechanism in the range of 5–300 K [22]. The magnetic properties at low temperature of Pr-Ce-Fe-B magnet have been studied [23]. In order to prompt the usage of high-abundance RE elements in Pr-Fe-B based magnets, it is necessary to further clarify the specific coercivity mechanism.

In this study a series of Ce-(Y)-Pr-Fe-B films were prepared by magnetron sputtering. By combination first-order reversal curve (FORC) and micromagnetic theory, the magnetization reversal process and coercivity mechanism were investigated.

## 2. Materials and Methods

Targets with compositions of nominal atomic ratio (Ce_0.3_Pr_0.7_)_18_Fe_71_B_11_ and (Ce_0.3_Y_0.1_ Pr_0.6_)_18_Fe_71_B_11_ were prepared by powder metallurgy method, and all raw materials are commercially available with 99.99% purity. Ta, as buffer layer and cover layer, is a commercial target material with a purity of 99.95%. The thin film samples were all prepared by high vacuum magnetron sputtering. The structures of all films were described as Si (001)/Ta (40 nm)/Ce-Pr-Fe-B (300 nm)/Ta (40 nm) (S1) and Si (001)/Ta (40 nm)/Ce-Y-Pr-Fe-B (300 nm)/Ta (40 nm) (S2). The base pressure was better than 2.5 × 10^−5^ Pa, while the sputtering rate of Ta was 2.148 Å/s, and the sputtering powers of (Ce_0.3_Pr_0.7_)_18_Fe_71_B_11_ and (Ce_0.3_Y_0.1_ Pr_0.6_)_18_Fe_71_B_11_ were maintained at 90 W. The calibration of the sputtering rate was carried out by weighing method. The buffer layer Ta was sputtered at room temperature, then the substrate was, heated to 500 °C under vacuum for preparing magnetic layers, and the whole sample was annealed at 630 °C for 20 min.

The analyses of phases and structures were carried out by using a Rigaku MiniFlex 600 X-ray diffractometer (XRD) (Rigaku Smartlab, Tokyo, Japan) with a grazing incidence of 1° in 2θ mode and single chromatic Cu kα1 radiation (λ = 0.154056 nm). The magnetic measurements at room temperature were carried out with a vibrating sample magnetometer (VSM) (Quantum Design, San Diego, CA, USA). The data for FORC and the micromagnetic analysis were obtained by a superconducting quantum interferometer device (SQUID) (Quantum Design, San Diego, CA, USA). The evolution of magnetic domains was performed by a physical property measurement system (PPMS) with a scanning probe microscope (SPM). All magnetic domains were obtained at room temperature by a magnetic force microscopy (MFM), and surface topography was obtained by an atomic force microscope (AFM) using tapping mode; domain contrast was measured by interleave scanning with a lift height of 80 nm. The detailed microstructure was investigated by a transmission electron microscopy (TEM) and a scanning transmission electron microscopy (STEM) on a Tecnai G2 F20 system (FEI, Hillsboro, OR, USA). The elemental distribution in the films was examined by X-ray energy dispersive spectroscopy (EDS).

## 3. Results and Discussion

**Figure 1:**
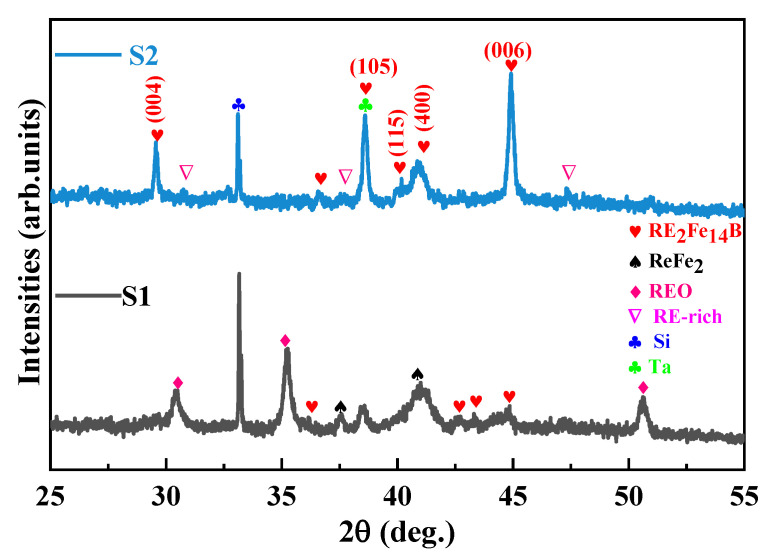
X-ray diffraction patterns of S1 and S2 film samples.

Figure 1 shows the XRD results of S1 and S2 films. Each film sample has diffraction peaks of RE_2_Fe_14_B phase. According to the position of the XRD peak, it is clear that the RE elements (Ce/Y/Pr) are all involved in the formation of the 2:14:1 type main phase. As compared with the result of the S1 sample, the strongest reflection is associated with the 2:14:1 tetragonal phase, such as (004), (105), and (006) peaks, so there is a better c-axis orientation in the S2 sample. The addition of Y leads to the inhibition of REO, REFe_2_ phases, and the appearance of RE-rich intergranular phases.

**Figure 2:**
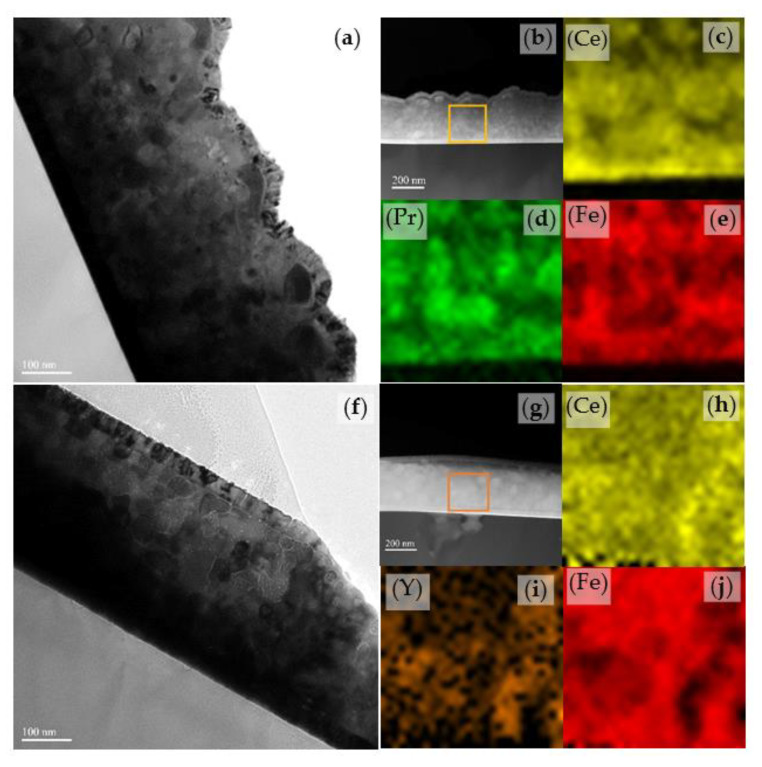
Cross-section bright-field TEM images of (**a**) S1 sample and (**f**) S2 sample; (**b**,**g**) are corresponding HADDF images of the S1 and S2 samples; the elemental concentration distribution mappings of (**c**) Ce, (**d**) Pr, (**e**) Fe of selection region in (**b**); the elemental concentration distribution mappings of (**h**) Ce, (**i**) Y, (**j**) Fe obtained from the selection square region in (**g**).

The microstructure and element distribution are further studied, as shown in Figure 2. The surface of the S2 sample is relatively flatter than that of the S1 sample. The bright contrasts are grain boundaries and the dark contrasts are grains of 2:14:1 matrix phase [16]. An obvious boundary is observed between the main phase and the grain boundary phase in the S2 sample. The Fe element shows the distribution of RE_2_Fe_14_B grains [24]. Therefore, from the element distribution, it is concluded that Y enters the 2:14:1 main phase. 

**Figure 3:**
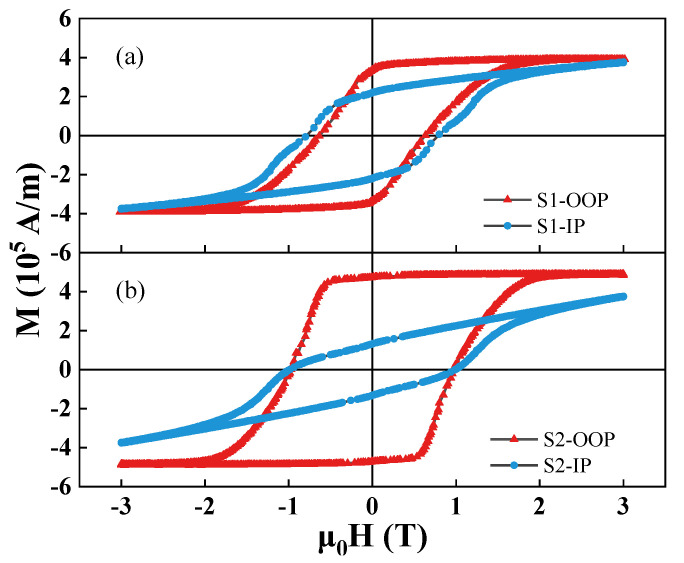
Hysteresis loops of (**a**) S1 sample and (**b**) S2 sample, in out-of-plane (OOP) and in-plane (IP) directions.

**Figure 4:**
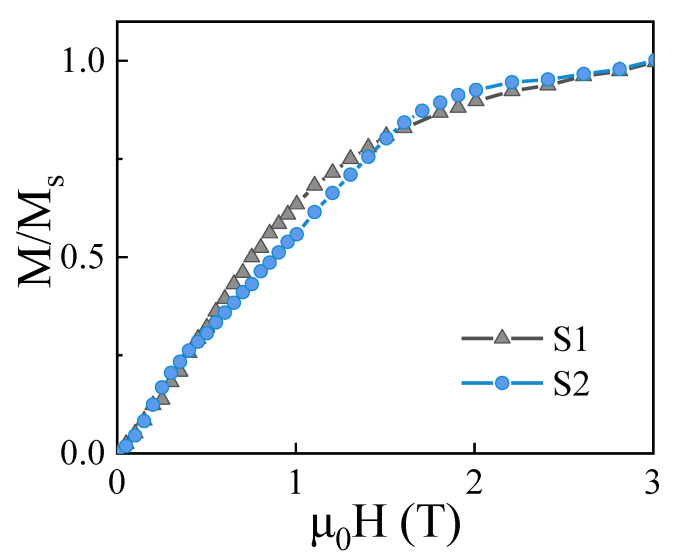
Normalized initial magnetization curves of both S1 and S2 samples.

The magnetic hysteresis loops and initial magnetization curves for both S1 and S2 samples measured at room temperature are given in Figure 3 and Figure 4. As shown in Figure 3b, the S2 sample has better perpendicular magnetic anisotropy (PMA) by comparing the difference of out-of-plane (OOP) and in-plane (IP) loops. It is seen from Figure 3a,b that the coercivity increases from 0.63 T to 0.97 T in the OOP direction. The OOP is the c-aixs direction of films. It can be concluded that the S2 sample has a better c-axis orientation. Meanwhile, the PMA and the squareness of hysteresis loop are also enhanced. The improvement of magnetic properties can be attributed to the following three aspects: an increase in the formation of the RE_2_Fe_14_B-type main phase with the enhanced c-axis orientation, a decrease in the REFe_2_ and REO phases, and a more orderly arrangement between hard magnetic main phase and other phases. Figure 4 shows the initial magnetization curves of the S1 and S2 samples. According to the shapes of initial magnetization curves, the coercivity mechanisms of the S1 and S2 samples belong to a mixture of nucleation and pinning mechanisms.

**Figure 5:**
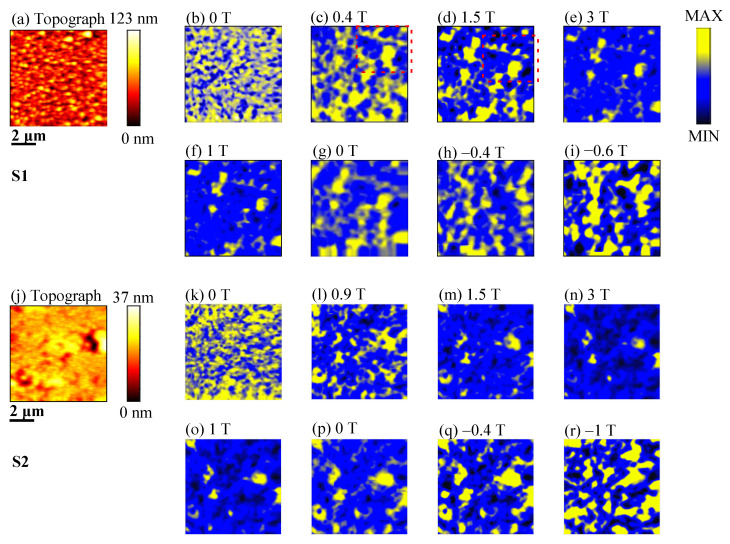
MFM images during the initial magnetization and the demagnetization process: (**a**) AFM topography of the S1 sample; (**b**–**i**) MFM images for the S1 sample; (**j**) AFM topography of the S2 sample; (**k**–**r**) MFM images of the S2 sample. All MFM images are obtained from the same area, which is consistent with the topography region.

Figure 5 illustrates the magnetic domain patterns of the S1 and S2 samples at room temperature during the initial magnetization and demagnetization processes, in which a magnetic field is applied perpendicular to the plane of the film. Blue and yellow domains represent the upward and downward magnetization orientations, respectively. During the initial magnetization process, for the S1 sample, it is obvious that the domain movement occurs from 0.4 to 1.5 T, as shown in the red square region in Figure 5c,d. From 1.5 to 3 T, as shown in Figure 5d,e, the blue domain expands while the yellow domain shrinks. For the demagnetization process from 3 to 1 T, as shown in Figure 5e,f, the domain contrast remains unchanged. When the applied field is absent in Figure 5g, the reversal domain begins to nucleate. From −0.4 to −0.6 T, as shown in Figure 5h,i, the yellow domain expands while the blue domain shirks. For the S2 sample, when the external magnetic field increases from 0 to 0.9 T, as shown in Figure 5k,l, a new domain distribution indicates an obvious displacement of domain wall. Then, from 0.9 to 3 T, as shonw in Figure 5l,n, the blue domain expands and the yellow domain shrinks. At the saturation state of 3 T, as shown in Figure 5n, some residual domains still exist, which is caused by the defects of microstructures or nonmagnetic phases in the samples [25]. For the demagnetization process from 3 to 1 T, as shown in Figure 5n,o, the basically unchanged domain contrast is consistent with the hysteresis loop, as shown in Figure 3b, meaning that the magnetization state is maintained in a saturated state. From 0 to −0.4 T, as shown in Figure 5p,q, the domain contrast remains unchanged. The yellow domain expands with further changing of the magnetic field to −1 T, as shown in Figure 5r.

The in situ analysis of domain evolution can help us understand the coercivity mechanism. It is seen that the magnetic domains of the two samples have formed a new distribution when the applied field is near 1.5 T, as shown in Figure 5d,m of the initial magnetization process. If the magnetization behavior is controlled only by the pinning mechanism, a new domain distribution is not formed, due to the large resistance of domain wall movement. In addition, from 1.5 to 3 T, the expansion of blue domain and the contraction of yellow domain indicate that a large external field is needed to reach the saturation state. Therefore, the pure nucleation mechanism is not the only dominant factor. From the analysis above, the coercivity mechanism is a mixed mechanism.

In order to distinguish the dominant mechanism for coercivity, it is necessary to further investigate by using micromagnetic theory. According to the theory, when the coercivity is determined by the nucleation mechanism, it can be processed with the following formula:

(1) $$H_{C}/M_{S}=\alpha H_{a}/M_{S}-N_{eff}$$

where, $H_{C}$, $H_{a}$, and $M_{S}$ are coercivity, anisotropy field, and saturation magnetization, respectively. The data of $H_{a}$ of Y_2_Fe_14_B, Ce_2_Fe_14_B, and Pr_2_Fe_14_B are obtained from reference [10]. The anisotropy fields are averaged according to the atomic ratio of RE elements in the targets. *N_eff_* defined as the local effective demagnetization factor is a microstructural coefficient, which describes the demagnetization effect resulting from grain surfaces and volume charges. $N_{eff}M_{S}=-H_{d}+2\pi M_{S}$, and the *H_d_* is demagnetizing field. $H_{d}=-N_{m}M_{S}-N_{g}M_{S}$$+N_{st}M_{S}$, where *−N_m_M_s_* is the macroscopic demagnetization field due to external surface charges, *−N_g_M_s_* is the magnetic demagnetization field induced by the magnetic charges on the surface of the magnetic grains in the magnet, and *N_st_M_s_* is the structural demagnetization field induced by the nonmagnetic grains or holes in the magnet. In addition, *α* is the microstructure parameter, $\alpha =\alpha_{K}\alpha_{\varphi }$; the coefficient $\alpha_{\varphi }$ is related to the crystal grains deviating from the c-axis, expressing a decrease in the nucleation field caused by misaligned crystal grains; $\alpha_{K}$ is related to the nonuniform defect region, indicating a decrease in the nucleation field due to the grain damage on the surface of the magnet and the imperfect internal grain; and the local effective demagnetization factor $N_{eff}$ is related to local demagnetization fields near sharp edges and corners of polyhedral grains. Then, $\alpha_{K}$ and $\alpha_{\varphi }$ can be expressed by the following formulas:

(2) $$\alpha_{\varphi }=\frac{1}{\cos\varphi }\frac{1}{{\left(1+\tan^{2/3}\varphi \right)}^{3/2}}\left(1+\frac{2K_{2}}{K_{1}}\frac{\tan^{2/3}\varphi }{1+\tan^{2/3}\varphi }\right)$$

(3) $$\alpha_{K}=1-\frac{1}{4\pi^{2}}\frac{\delta_{B}^{2}}{r_{0}}{\left[-1+{\left(1+\frac{4\Delta Kr_{0}^{2}}{A}\right)}^{1/2}\right]}^{2}$$

where φ is the angle between the external field and the c-axis; $\delta_{B}$, ΔK, A, and $r_{0}$ are defined as the width of Bloch domain wall, the reduction of magneto-crystalline anisotropy constant in defect region, the exchange constant, and the half width of the planar defect region, respectively. Generally, the nucleation mechanism is examined in the following two situations [26,27,28]:

(1) If the magnetization reversal process is uniform, in other words, it is controlled by the uniform reversal of the magnetic moment, then, a linear relationship between $H_{C}/M_{S}$ and $H_{a}/M_{S}$ exists within the entire temperature range.

(2) Considering that the magnetic particles are strongly coupled and the anisotropy axis is not strictly along the c-axis, one reversed magnetic moment would lead to the joint reversal of the surrounding magnetic moments. In this case, it is necessary to consider the influence of the existence of strong misorientation grains on coercivity, and $\alpha =\alpha_{\varphi }^{\min}.$

**Figure 6:**
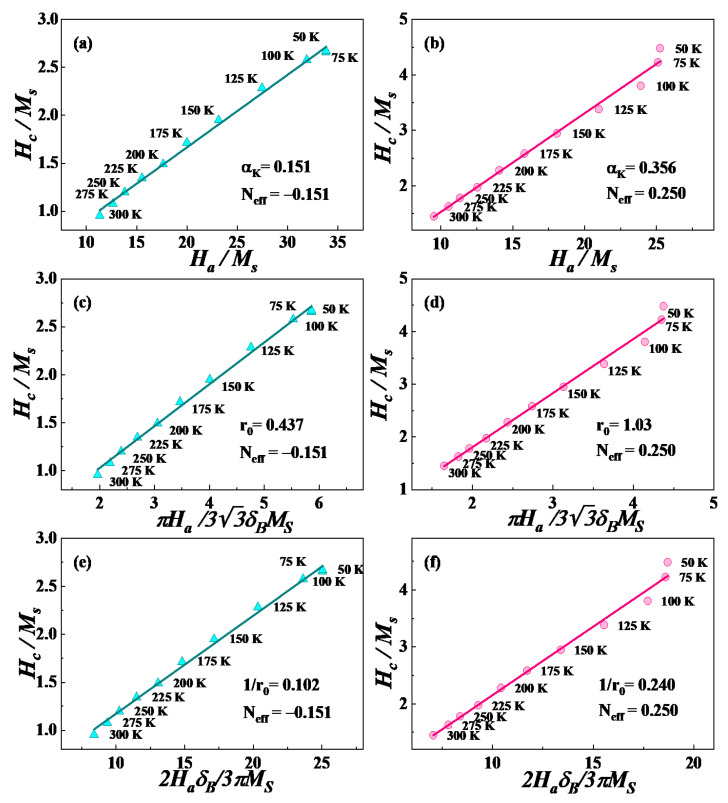
(**a**,**b**) Plots of $H_{C}/M_{S}$ versus $H_{a}/M_{S}$ for the S1 and S2 samples for testing the nucleation mechanism where the magnetization process is uniform; (**c**,**d**) the plots of $H_{C}/M_{S}$ versus $\pi H_{a}/3\sqrt{3}\delta_{B}M_{S}$ for the S1 and S2 samples for testing the pinning mechanism where the pinning centers are thin inhomogeneities; (**d**–**f**) the plots of $H_{C}/M_{S}$ versus $2H_{a}\delta_{B}/3\pi M_{S}$ for the S1 and S2 samples to test the pinning mechanism in which the pinning centers are extended planar faults.

In Figure 6a,b, a good linear relationship is clearly observed in a wide temperature range from 50 to 300 K, which shows that the magnetization reversal process of both samples is uniform. According to micromagnetic theory, the coercivity mechanism should be a nucleation mechanism if $\alpha_{K}>0.3$, while it may be determined by a pinning mechanism or by a nucleation mechanism when $\alpha_{K}<0.3$ [26]. In addition, the larger $\alpha_{K}$ value means a stronger nucleation field. Here, we choose $\alpha_{\varphi }^{\min}=0.5$ [29] to estimate $\alpha_{K}$. The fitting results show that for the S1 and S2 samples, $\alpha_{K}$ are 0.151 and 0.356, respectively. Therefore, the nucleation mechanism plays a leading role in the S2 sample, however, for S1, the coercivity mechanism remains to be further analyzed.

If the coercivity mechanism is dominant by a pinning mechanism, it also needs to consider two different situations [26]:

(a) In the case of thin inhomogeneities ($2r_{0}<\delta_{B}$), the coercivity field satisfies the following relation:
  (4) $$\frac{H_{C}}{M_{S}}=\frac{\pi }{3\sqrt{3}}\frac{r_{0}}{\delta_{B}}\frac{H_{a}}{M_{S}}-N_{eff}$$
(b) On the contrary, if the domain wall is pinned by extended planar defects ($2r_{0}>>\delta_{B}$), the coercivity field satisfies the following expression:
  (5) $$\frac{H_{C}}{M_{S}}=\frac{2}{3\pi }\frac{\delta_{B}}{r_{0}}\frac{H_{a}}{M_{S}}-N_{eff}$$

Figure 6c,d shows the results of $H_{C}/M_{S}$ and $\pi H_{a}/3\sqrt{3}\delta_{B}M_{S}$ of the S1 and S2 samples, from 50 to 300 K. It is clear that there is also a linear relationship in the whole temperature range. Meanwhile, the values of $r_{0}$ obtained from the fitting results are 0.437 and 1.03 nm, respectively. The maximum value of $2r_{0}$ is 2.06 nm, while the width of the domain wall of the Pr-Fe-B magnet at room temperature is 3.49 nm [30], hence, the prerequisite ($2r_{0}<\delta_{B}$) is satisfied, indicating that the narrow plane defects play a certain role in determining coercivity. 

Figure 6e,f plots the results of $H_{C}/M_{S}$ and $2H_{a}\delta_{B}/3\pi M_{S}$ of samples from 50 to 300 K, respectively. A linear relationship also exists over the entire temperature range. The values of $2r_{0}$ obtained from the fitting results are 19.6 and 8.34 nm, respectively. Only the wide planar defect shown in Figure 6e contributes to coercivity. For the S2 sample, the width does not meet the prerequisite ($2r_{0}>>\delta_{B}$) [31], indicating that the inhomogeneous planar regions do not play a certain role in determining coercivity. For the S1 sample, the negative value of *N_eff_* is related to the large structural demagnetization caused by more nonmagnetic phases and other substances in the S1 sample [31]. Another reason might be the simulated value being a local effective demagnetization factor but not the demagnetization factor *N*. In the S2 sample, the value of *N_eff_* is larger than that of the S1 sample, which may be related to the reduction of nonmagnetic phases in the sample and the microstructure with obvious boundaries between the main phase and the grain boundary phase (as shown in Figure 2). It is concluded that the coercivity mechanisms are a mixture type. In the S1 sample, the dominant mechanism cannot be distinguished from the above results; for the S2 sample, the nucleation mechanism is dominant.

In order to further study the magnetization reversal process and the magnetic interaction between the grains, the FORC method was applied. The magnetization of the sample was first saturated, then, the external magnetic field was reduced to the reversal field ($H_{r}$), and then the field measurement was carried out from $H_{r}$ to saturation field. In this way, hundreds of minor loops were obtained to form the FORC diagram. The magnetization is $M\left(H,H_{r}\right)\left(H>H_{r}\right)$, where *H* is the applied magnetic field. The FORC distribution parameter is defined as [32,33]:

(6) $$\rho =-\frac{1}{2}\frac{\partial^{2}M\left(H,H_{r}\right)}{\partial H\partial H_{r}}$$

**Figure 7:**
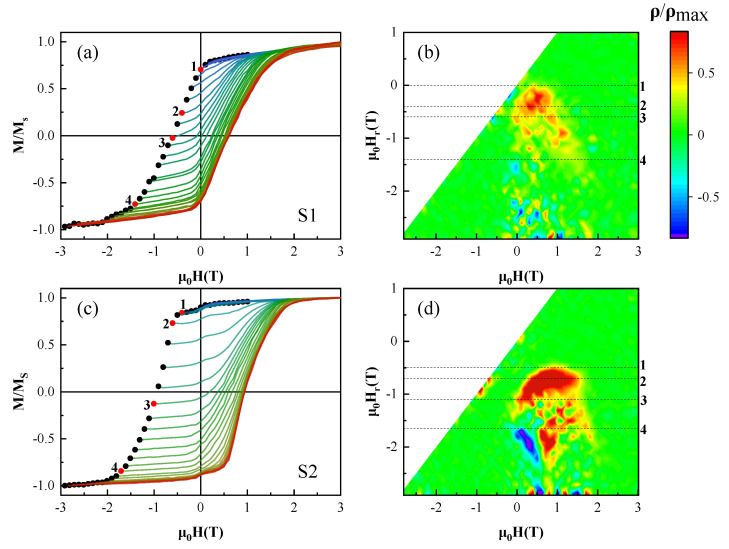
(**a**,**c**) FORC (normalized to 1) along OOP direction of the S1 and S2 samples; (**b**,**d**) are the contour plots of the $\mu_{0}H_{r}$ and $\mu_{0}H$ dependence of FORC function distribution, respectively.

The completely reversible component of magnetization is eliminated by the second order derivative. In other words, the distribution of ρ=0 indicates that the magnetization process is reversible [34]. Otherwise, the distribution of ρ≠0 indicates that the magnetization process is irreversible. Figure 7 represents the corresponding FORC graph. The left side of the graph shows the normalized FORC, and the graphs on the right side are the corresponding contour graph. 

In Figure 7d, before Point 1, the value of ρ is close to zero, and the magnetization process is reversible, during this part, the movement of magnetic domain occurs. Before the reversal, the magnetic domain generally moves to keep the magnetic energy at the lowest state, Figure 5e,f,n,o also embodies this feature. Between Points 1 and 3, nucleation of the reversal domain wall occurs, and their successive movements promote the reversal. The peak of ρ occurs at Point 2. From Point 3, ρ gradually goes to the plane of ρ = 0. Starting from Point 4, FORC presents two nonzero tails and begins to show a negative ρ value. The tail of positive value indicates that the residual domain in the previous stage begins to annihilate, whereas nonzero negative tail means that the magnetization state begins to develop towards negative saturation. Similar phenomena also occur in both samples under different applied magnetic fields. As compared with the FORC results, for the S2 sample, the starting magnetic field of the irreversible magnetization process increases, and the nonzero tail is more obvious. Meanwhile, the positive and negative trailing pair on the bottom of Figure 7d corresponds to the situation that the saturation magnetization remains unchanged but the coercivity increases on the minor FORC loops, as shown in Figure 7c, which is a characteristic of the typical nucleation mechanism. Based on the above analysis, it can also be determined that the S2 sample is controlled by the nucleation mechanism at room temperature. Therefore, the reversal is initiated by the formation of the reversal domain wall and their successive movements. In addition, due to other optimizations brought by adding Y, the dominant coercivity mechanism gradually becomes a nucleation mechanism.

The FORC method can be used to characterize the magnetic interaction and coercivity distribution by transforming the coordinate system as follows:

(7) $$\mu_{0}H_{u}=\left(\mu_{0}H+\mu_{0}H_{r}\right)/2$$

(8) $$\mu_{0}H_{c}=\left(\mu_{0}H-\mu_{0}H_{r}\right)/2$$

**Figure 8:**
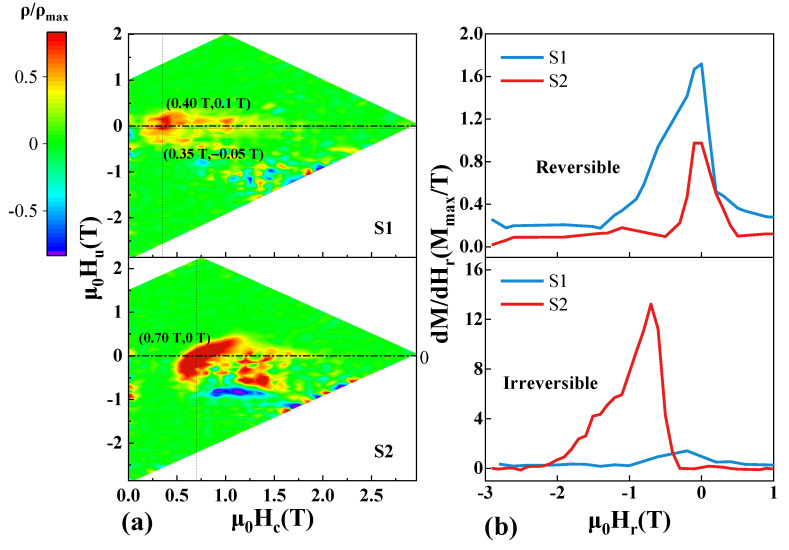
(**a**) FORC distributions of the S1 and S2 samples, in the $\mu_{0}H_{u}$ and $\mu_{0}H_{c}$ coordinate system; (**b**) extracted reversible and irreversible distributions, which correspond to the S1 and S2 samples.

In this coordinate system, the distribution of peak along $\mu_{0}H_{c}>0$ represents the distribution of coercivity, without considering the interaction between particles. The $\mu_{0}H_{u}$ represents the distribution of mean interaction filed. The distribution function in the coordinate system is shown in Figure 8a. The maximum value of the distribution function is $\rho_{\max}$, which depends on the interaction between ferromagnetic particles. Meanwhile, for the S1 and S2 samples, the values of $\rho_{\max}$ are 1.27 × 10^−11^ and 2.12 × 10^−11^, respectively. The value of the S2 sample is almost twice that of the S1 sample, meaning the interaction is strengthened.

As shown in Figure 8a, along the axis of $\mu_{0}H_{c}$, the phenomenon of peak separation is found, because different RE elements participate in the formation of the 2:14:1 main phase, which leads to the inhomogeneous magnetization reversal, although the grains are coupled on the whole. In addition, for the S1 sample, there are two maxima along the axis of $\mu_{0}H_{u}$, which is related to the different sites of nucleation and annihilation of phases. The peak corresponding to the positive interaction field is related to the rapid development of the stripe domain, while the peak corresponding to the negative value is associated with domain annihilation. This feature is consistent with the in situ magnetic domain evolution, shown in Figure 5f–h, in the S1 sample [34,35]. The high degree of disorder in the sample and the low exchange interaction results in this phenomenon.

Along the direction of the $\mu_{0}H_{u}$ axis, when the maximum value of ρ is distributed in the positive direction of $\mu_{0}H_{u}$, the average interaction is a dipole interaction. If the distribution is biased towards the negative direction of the $\mu_{0}H_{u}$ axis, the mean interaction tends to be an exchange interaction [36]. For the S1 sample, both the exchange interaction and dipole interaction coexist. However, the maximum value located below $\mu_{0}H_{u}$ indicates that the exchange interaction is stronger. For the S2 sample, the maximum value is on the axis $\mu_{0}H_{u}=0$, indicating that the exchange and dipole interaction are in the same order of magnitude, but the exchange interaction is slightly larger through the deflection direction of the overall FORC distribution [36]. 

The data of FORC reflects the reversible and irreversible processes in the whole magnetization reversal process [33,37,38]. Figure 8b shows the distribution curves of the reversible and irreversible parts separated from the FORCs data. The separation of the reversible and irreversible processes has been performed near the descending branch of the major loop by monitoring the magnetization change versus $H_{r}$ in the original FORC [39]. In mathematical form, the reversible/irreversible parts are represented by the following formulas:

(9) $$dM^{rev}\left(H_{r}\right)=\underset{H\to H_{r}}{\lim}\left[M\left(H_{r},H\right)-M\left(H_{r}\right)\right]$$

(10) $$dM^{irr}\left(H_{r}\right)=\underset{H\to H_{r}}{\lim}\left[M\left(H\right)-M\left(H_{r},H\right)\right]$$

Since the reversible components in the magnet are mainly provided by the soft magnetic phase, while most irreversible components are provided by the hard magnetic phase, the reversibility and irreversibility can be reflected in the magnetization reversal process. The reversible component is mainly distributed near $\mu_{0}H_{r}=0$, while the irreversible curve is mainly distributed near the coercivity. For the S2 sample, the initial distribution field of the irreversible part is larger, and the position of the extreme value is high, and the distribution range is wider. Then, the corresponding reversible and irreversible proportions are calculated. The irreversible proportions of the S1 and S2 samples are 54.8% and 94.9%, respectively. Another method to obtain the reversible proportion has also been applied [40], and the results are similar to the previous results. It is concluded that the S2 sample has a large irreversible ratio, which also indicates that more 2:14:1 main phase and good microstructure are formed in the S2 sample, qualitatively.

In short, the magnetization reversal process can be better understood through the FORC distribution and the in situ domain evolution results. In the S1 and S2 samples, exchange interaction is mainly the mean interaction. However, in the S1 sample, the microstructure is chaotic, and dipole interaction also exists. For the S2 sample, the proportion of irreversible components is relatively high.

## 4. Conclusions

Compared with the sample with a structure of Ta (40 nm)/(Ce_0.3_Pr_0.7_)_18_Fe_71_B_11_ (300 nm)/Ta (40 nm) (S1), the addition of Y and a subsequent heating treatment in the sample with structure of Ta (40 nm)/(Ce_0.3_Y_0.1_ Pr_0.6_)_18_Fe_71_B_11_ (300 nm)/Ta (40 nm) (S2) lead to the formation of less REFe_2_ and REO phases, and RE_2_Fe_14_B with stronger c-axis orientation. Meanwhile, the S2 sample exhibits more comprehensive magnetic properties. By using the initial magnetization curve, MFM and FORC distribution, it is found that the coercivity mechanism in the S2 sample is a mixture mechanism, but dominated by a nucleation mechanism. The magnetization reversal process can be better understood by combining the FORC distribution with the in situ domain evolution results. The irreversible reversal is caused by the nucleation of the reversal magnetic domains and their successive movements. The results are beneficial for understanding the coercivity mechanism and magnetization reversal process of permanent magnets with high-abundance RE elements, and promote the application of RE elements, Ce and Y, in the permanent magnet industry.

## Data Availability

The data that supports the findings of this study are available within the article.
